# Nutraceutical Potential of Leaf Hydro-Ethanolic Extract of the Edible Halophyte *Crithmum maritimum* L.

**DOI:** 10.3390/molecules26175380

**Published:** 2021-09-04

**Authors:** Aymen Souid, Clara Maria Della Croce, Stefania Frassinetti, Morena Gabriele, Luisa Pozzo, Marco Ciardi, Chedly Abdelly, Karim Ben Hamed, Christian Magné, Vincenzo Longo

**Affiliations:** 1 Institute of Biology and Agricultural Biotechnology (IBBA), CNR, Research Area of Pisa, Via Moruzzi 1, 56124 Pisa, Italy; souid.ayman@gmail.com (A.S.); clara.dellacroce@ibba.cnr.it (C.M.D.C.); frassinettistefania@gmail.com (S.F.); morena.gabriele@ibba.cnr.it (M.G.); marco.ciardi@ibba.cnr.it (M.C.); vincenzo.longo@ibba.cnr.it (V.L.); 2 Laboratoire des Plantes Extrêmophiles, Centre de Biotechnologie de Borj Cedria, BP 901, Hammam Lif 2050, Tunisia; chedly.abdelly@cbbc.rnrt.tn (C.A.); karimbenhamed2016@gmail.com (K.B.H.); 3 EA7462 Géoarchitecture_Territoires, Urbanisation, Biodiversité, Environnement, Université de Brest, 6 Avenue Victor Le Gorgeu, CS 93837, CEDEX 3, 29238 Brest, France; Christian.Magne@univ-brest.fr

**Keywords:** *Crithmum maritimum* L., antioxidant capacity, phytochemicals, antibacterial activity, antibiofilm activity, antimutagenic effect, functional food

## Abstract

Aromatic halophytes represent an exceptional source of natural bioactive compounds for the food industry. *Crithmum maritimum* L., also known as sea fennel, is a halophyte plant colonizing cliffs and coastal dunes along Mediterranean and Atlantic coasts. It is well known to produce essential oils and polyphenols endowed with antioxidant and biological effects. The present work reports the phytochemical profile, as well as antioxidant, antimicrobial and antimutagenic properties of *C. maritimum* leaf hydro-alcoholic extract. From LC-ESI-MS analysis, eighteen phenolic compounds were depicted in sea fennel extract and the amount of total phenolic content exceeds 3% DW. Accordingly, *C. maritimum* extract showed strong antioxidant activities, as evidenced by in vitro (DPPH, ORAC, FRAP) and ex vivo (CAA-RBC and hemolysis) assays. An important antimicrobial activity against pathogenic strains was found as well as a strong capacity to inhibit *Staphylococcus aureus* (ATCC 35556) biofilm formation. Sea fennel extracts showed a significant decrease of mutagenesis induced by hydrogen peroxide (H_2_O_2_) and menadione (ME) in *Saccharomyces cerevisiae* D7 strain. In conclusion, our results show that *C. maritimum* is an exceptional source of bioactive components and exert beneficial effects against oxidative or mutagenic mechanisms, and pathogenic bacteria, making it a potential functional food.

## 1. Introduction

The importance of nutraceuticals, functional food and other natural diet compounds extracted from plants has been well documented in relation to health promotion and illness risk reduction. Many recent studies have provided a clear insight on the physiological mechanisms of the effects of bioactive molecules and natural compounds on human health security [1,2,3]. In addition, the human population has started to pay attention for and to consume these spontaneous plants, including them in new recipes, and increasing their demand and availability in the market. Beyond their known value as ornamentals in gardens or flower bouquets, their rediscovered organoleptic characteristics give these plants a gastronomic potential.

Natural products such as polyphenols, enzymes and vitamins obtained from aromatic plants have been of great interest for the food and pharmaceutical industries due to their important biological properties [4]. Amongst bioactive molecules-bearing plants, the halophytes are recognised as a source of constituents with both medicinal and nutritional value [5]. In the last decades, there has been an increasing use of halophytic plants, including *Salicornia herbacea*, *Inula crithmoides*, *Portulaca oleracea* and *Crithmum maritimum*, as non-conventional foods [2,6,7,8]. 

Sea fennel (*Crithmum maritimum* L., Apiaceae) is a spontaneous aromatic plant and the sole species of the genus *Crithmum*. This facultative and perennial halophyte is widely distributed along Mediterranean coasts of Italy, Greece, Tunisia, Spain as well as Atlantic shorelines of Portugal and France [2,9]. Its aerial parts are edible and can be consumed fresh as salad vegetables, or pickled [8,9,10]. Actually, sea fennel leaves produce macro and micronutrients, dietary fibers, vitamins, polyunsaturated fatty acids and useful secondary metabolites, providing health benefits and disease prevention. Therefore, *C. maritimum* may be considered as a promising wild edible plant for the future as it encloses interesting bioactive natural substances that may be used as nutraceuticals or agro-food supplements [2,11].

In recent years, there has been an increased interest in natural antimicrobials since microorganisms are involved in the deterioration of food or cosmetic matrices, and a wide range of diseases. Some plant species are a rich source of natural compounds with antimicrobial properties, which are able to prevent the growth of foodborne pathogens [12]. Several studies demonstrated the antimicrobial activity of dietary secondary metabolites like polyphenols [13,14]. Along, pathogens involved in foodborne diseases or food processing plant contamination are often capable to adhere and form biofilms. However, literature about plant constituents with antifouling capacity is scarce, particularly concerning halophytes.

It is well known that anti-mutagens play an important role in preventing the cell damage that can be induced by oxidative agents. Accordingly, in vitro bioassays have been developed to study the protective antimutagenic effects of food derivatives and food extracts [15]. Here, the antimutagenic properties of *C. maritimum* extracts were evaluated using the D7 strain of *Saccharomyces cerevisiae* yeast. 

The aim of this study was to increase the knowledge on the effect of *C. maritimum* leaf hydro-ethanolic extract on redox cellular status and to evaluate its antioxidant, antimicrobial and antibiofilm activity, as well as the antigenotoxic properties. For this purpose, the antioxidant effect of *C. maritimum* was evaluated by in vitro tests and on ex vivo human erythrocytes, the antimicrobial activity was investigated against pathogenic strains, and the antimutagenic effect was studied in the yeast *S. cerevisiae* D7 strain.

## 2. Results and Discussion

In recent years, a great attention has been given to the qualitative and quantitative characterization of secondary metabolites and their antioxidant activities in different plant extracts, and specifically spontaneous plants. In this regard, it has been reported that antioxidant compounds, particularly phenolics, are recovered in methanol or acetone extracts. Hence in this study, phenolic compounds and their biological activities were measured in green hydro-ethanolic extract of *C. maritimum* leaves, using different techniques and bioassays.

### 2.1. Phenolic Contents and Antioxidant Activities of C. maritimum Extract

#### 2.1.1. Phenolic Compounds and Phytochemical Profile 

The total contents of polyphenols and of phenolic classes in sea fennel leaf extract were evaluated and are presented in Table 1. Thus, *C. maritimum* leaves contained 31.7 mg GAE g^−1^ DW of polyphenols and 25.6 mg CE g^−1^ DW of flavonoids, including 17.3 mg QE g^−1^ DW of flavonols. The hydro-ethanolic extract exhibited a higher concentration of total polyphenols compared to that found before in acetonic or methanol extracts of the same species sampled at the same place [6]. Accordingly, the amount of these phytochemicals has been shown to vary significantly depending on the extraction solvent [8,16,17,18]. Moreover, many studies showed that sea fennel plants harvested from different climatic areas or at different development stages exhibit contrasting phenolic contents and compounds [1,2,6,8]. As for the condensed tannins contents, *C. maritimum* extract contained 0.97 mg CE g^−1^ DW. Similar tannin levels were found in sea fennel plants collected from different biotopes [2,19].

**Figure 1:**
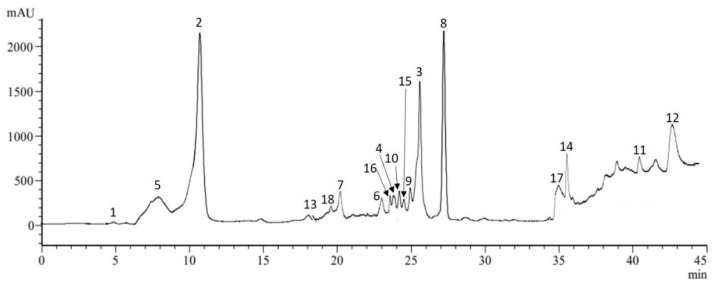
LC-ESI-MS analysis (280 nm) of soluble phenolic compounds in hydro-ethanolic extract of *C. maritimum leaves*. Peak numbers refer to the standard compounds mentioned in Table 2.

The phytochemical profile of sea fennel leaves was further investigated by LC-ESI-MS analysis to identify the individual phenolic constituents. Eighteen compounds were identified in the hydro-ethanolic extract, among which eight phenolic acids and nine flavonoids including four flavonols. Altogether, these eighteen constituents totalized 25.03 mg/g DW (Table 2 and Figure 1).

Noteworthy, cirsiliol was identified here for the first time in sea fennel. Even, as far as we know, this flavonoid has only been reported hitherto in Asteraceae, Lamiaceae and Malvaceae families [20,21,22], but not in Apiaceae. Besides, among the phenolic acid class, eight compounds were detected among which the most abundant were chlorogenic (7.25 mg/g DW), neochlorogenic (2.03 mg/g DW), trans-ferulic (1.41 mg/g DW) and cryptochlorogenic (1.17 mg/g DW) acids. Regarding the flavonoid class, rutin and cirsiliol were the major compounds detected (1.75 and 1.31 mg/g DW, respectively), along with quercetrin and hyperoside as the most abundant flavonols. Previous reports also showed the abundance of chlorogenic acid and its components, quinic acid and ferulic acid, in different sea fennel extracts (methanolic extract, infusion and decoction) [1,6,8]. Our investigation confirmed the presence of six hydroxycinnamic acids and of flavonoids reported by these authors, with the presence of rutin, hyperoside, quercetrin and kaempferol at high amounts. According to these authors, the sea fennel is among the highest phenolic containing species within the Apiaceae family. In particular, chlorogenic acid and its isomers appeared as major compounds, making the leaves of sea fennel a valuable alternative source of chlorogenic acids (and other phenolic compounds) for food industry. According to Santana-Gàlvez et al. [23], these phytochemicals are a promising nutraceutical and food additive attending to their multifunctional properties. Besides, chlorogenic acid has several reported biological activities including antioxidant, antimicrobial and anti-carcinogenic properties [23,24].

#### 2.1.2. In Vitro Antioxidant Properties

The antioxidant potential of *C. maritimum* leaf extract was assessed by three in vitro assays targeting the radical scavenging activity (DPPH), the antioxidant capacity (ORAC) and the metal-related antioxidant power (FRAP). Our results showed that sea fennel extract exhibited a strong antioxidant activity, with DPPH IC_50_ of 0.22 mg/mL, 15,835 ORAC units per gram of DW and a FRAP EC_50_ of 1.82 mg/mL (Table 1). Our results were in accordance with those found by previous authors using other extraction processes [2,6,8,19]. These antioxidant properties of sea fennel extract are likely related to its high level of phenolic compounds, particularly of chlorogenic acids. Indeed, the antioxidant capacity of plant extracts is highly associated to their phenolic content [25], and chlorogenic acid is a strong antioxidant compound often linked to the radical scavenging capacity [1,8]. Consequently, some plant products are important dietary sources of these bioactive compounds [2,26], the intake of which is associated with the prevention or amelioration of oxidative stress-related diseases, as a strategy to address such health challenges [6,11].

### 2.2. Biological Effects of C. maritimum on Human Erythrocytes 

The ex vivo antioxidant properties of *C. maritimum* leaf hydro-ethanolic extract were evaluated on human erythrocytes upon oxidative insult using the CAA-RBC assay and the hemolysis test. These cells, having neither nucleus nor mitochondria, represent a good cell-model system for the screening of antioxidant natural sources [27]. Thus, the antioxidant potential of sea fennel extract was evaluated in terms of cellular antioxidant activity and oxidative hemolysis inhibition in human erythrocytes exposed to the AAPH, a peroxyl radical generator causing oxidative stress, following 1-h pre-treatment with increasing doses of *C. maritimum* extract (0.05, 0.1 and 0.2 mg/mL).

**Figure 2:**
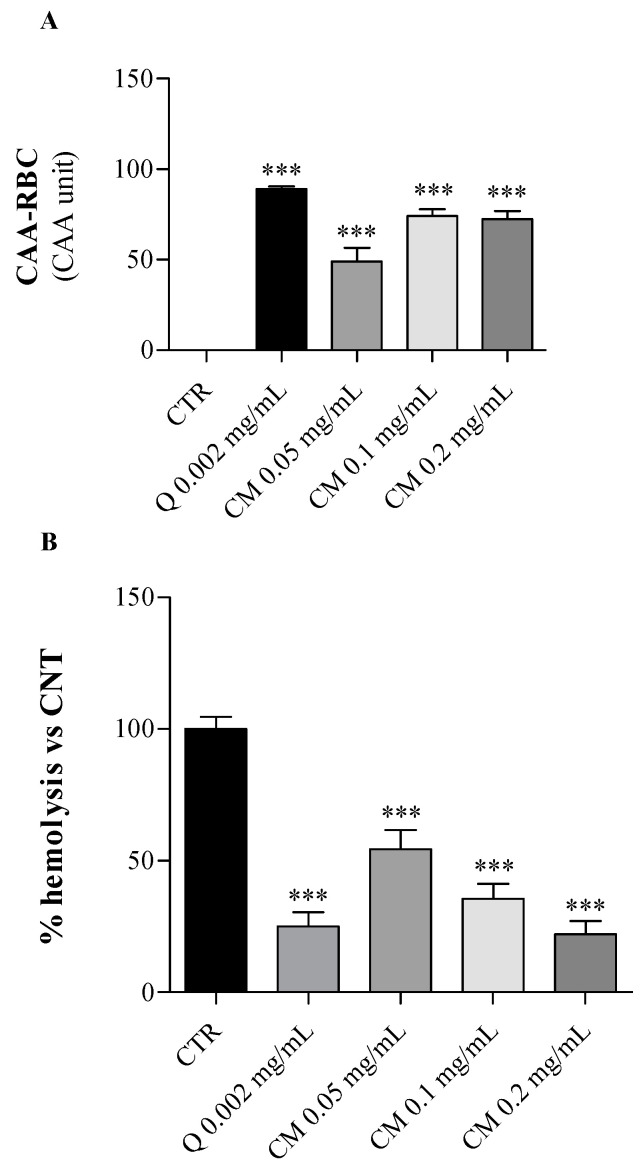
Effects of *C. maritimum* L. extract (CM) at increasing doses (0.05, 0.1 and 0.2 mg/mL) on human erythrocytes under oxidative insult. Quercetin (Q 0.002 mg/mL) was used as a reference standard. (**A**), CAA-RBC assay; (**B**), hemolysis test. Values are expressed as means ± standard deviation of 5 blood samples. *** Highly significant difference from control cells (CTR) (*p* ≤ 0.001).

As shown in Figure 2A, *C. maritimum* extract at all tested concentrations significantly increased (*p* < 0.001) the erythrocyte’s antioxidant activity compared to oxidized control cells only treated by AAPH (CAA = 0). Erythrocytes pre-treated with sea fennel extract at 0.1 mg/mL or more showed similar antioxidant activities to those treated with quercetin (0.002 mg/mL) used as a reference standard. 

Besides, the anti-hemolytic effect of *C. maritimum* was tested on human erythrocytes exposed to a strong oxidative insult (AAPH = 50 mM), which induces the erythrocyte hemolysis. Sea fennel extract pre-treatment of erythrocytes significantly lowered the AAPH-induced oxidative hemolysis (*p* < 0.001) in a dose-dependent manner (Figure 2B). The percentage of hemolysis inhibition ranged from 46% to 78% (at 0.05 and 0.2 mg/mL, respectively) compared to control erythrocytes treated with AAPH only (CTR, maximum hemolysis). The highest extract concentration induced similar hemolysis inhibition to quercetin pre-treatment (75%). These results confirm the general antioxidant potential of *C. maritimum* leaf, and these findings are in agreement with those previously obtained in different salt-tolerant plants [28]. Indeed, the halophytes *Limonium vulgare* and *L. delicatulum* exhibited a strong ability to protect erythrocytes from oxidative damages, and the former had the greatest anti-hemolytic effect and cellular antioxidant activity. 

### 2.3. Antimicrobial Activity of C. maritimum L. Extract

#### 2.3.1. Inhibition of Bacterial Growth

**Figure 3:**
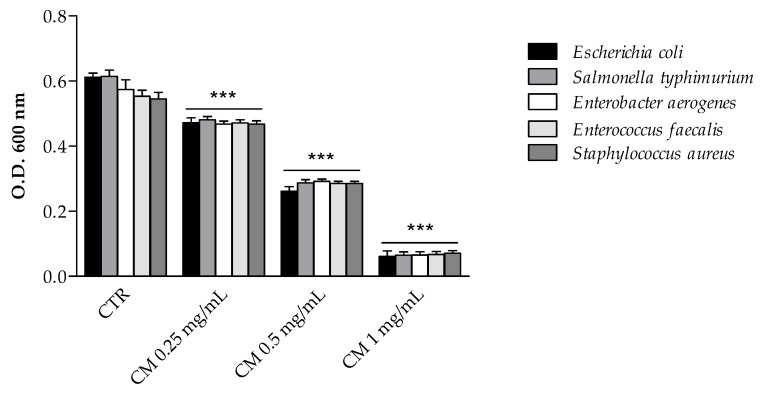
Antimicrobial activity (final O.D.) of *C. maritimum* L. extract (CM) (0.25, 0.50 and 1.00 mg/mL) against Gram negative bacteria (*Escherichia coli* ATCC 25922, *Salmonella enterica* ser. *typhimurium* ATCC 14028, and *Enterobacter aerogenes* ATCC 13048) and Gram positive bacteria (*Enterococcus faecalis* ATCC 29212 and *Staphylococcus aureus* ATCC 25923). Results are reported as mean values ± standard deviation (*n* = 3). *** Highly significant difference from negative control (CTR) (*p* ≤ 0.001).

The antimicrobial activity of sea fennel extract was first measured by evaluating the final growth of selected enteric bacterial strains in the presence of increasing doses of extract (Figure 3). *C. maritimum* extract, even at the lowest concentration (0.25 mg/mL), significantly inhibited the growth of every tested bacteria. The inhibitory effect on Gram-positive strains was similar to that on Gram-negative ones and appeared to be dose-dependent. The antimicrobial activity of plant phenolics has been extensively investigated against many different microorganisms [13,29]. The antimicrobial activity of *C. maritimum* extract may be related to its high content of chlorogenic and neochlorogenic acids, since both isomers have been shown to induce the disruption of the bacterial membrane, which changes the intracellular potential and leads to the death of bacteria [30]. Such antimicrobial activity of chlorogenic acid derivatives have been reported against a wide variety of bacteria including *Staphylococcus aureus*, *Streptococcus pneumoniae*, *Bacillus subtilis*, *Bacillus cereus*, *Escherichia coli, Enterococcus faecalis*, and *Salmonella typhimurium* [30,31,32,33]. Besides, *C. maritimum* extract showed a significant content of rutin and quercetin derivatives, which proved to be efficient inhibitors of the growth of several bacteria including *Escherichia coli*, *Staphylococcus aureus*, *Enterococcus faecalis* and *Pseudomonas aeruginosa* [29]. Therefore, sea fennel hydro-ethanolic extract is confirmed to have a great antimicrobial potential, although the authors at the moment have not yet determined the minimum inhibitory concentration (MIC) and the minimum bactericidal concentration (MBC), as it has also been reported for its essential oils [34,35].

#### 2.3.2. Biofilm Inhibition 

Pathogens involved in foodborne diseases or food processing plant contamination are often capable to adhere and form biofilms. These structures are organized communities of bacterial cells enclosed in a self-produced polymeric matrix, composed of polysaccharides, proteins and other organic components, adhering to inert or living surfaces. *Staphylococcus aureus* is a well-known pathogen living as biofilm in a wide variety of environments and represents a severe risk of food contamination.

**Figure 4:**
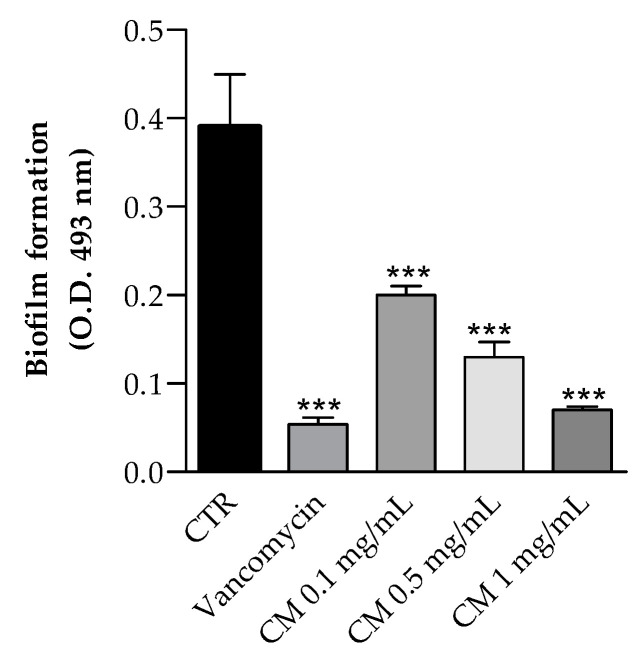
Effect of *C. maritimum* L. extract (CM) (0.25, 0.50 and 1.00 mg/mL) on *Staphylococcus aureus* (ATCC 35556) biofilm formation. Results are reported as means values ± standard deviation (*n* = 3). *** Highly significant difference from negative control (CTR) (*p* ≤ 0.001).

The effects of hydro-ethanolic *C. maritimum* extract on biofilm formation by *Staphylococcus aureus* ATCC 35556, a strong biofilm producer, was also investigated. As shown in Figure 4, *C. maritimum* extract at concentration of 0.1, 0.5, and 1 mg/mL significantly reduced the biofilm formation with an inhibition rate of 49%, 67%, and 82%, respectively. Górniak and collegues demonstrated that the ability to inhibit biofilm production by *Staphylococcus aureus* (ATCC 35556) is one of the mechanisms of action that allow the flavonoids to carry out the antimicrobial activit [36], and this may be one of the mechanisms underlying the antimicrobial activity of the hydro-ethanolic *C. maritimum* extract. To our knowledge, there are no results about the effect of hydro-alcoholic sea fennel extract of biofilm formation. Only Alves-Silva et al. [37] reported that the essential oil of *C. maritimum* has a strong effect on virulence factors of *C. albicans* and is able to inhibit biofilm formation by decreasing both the biomass and the cell viability. In our study, the inhibition of biofilm could be explained by the presence of chlorogenic, rutin, and quercetin in the extract, which were found to inhibit *Staphylococcus aureus* biofilm [32,38,39].

### 2.4. Antimutagenesis Assay in Yeast Cells

The eukaryotic *Saccharomyces cerevisiae* model D7 strain, obtained from the crossing of two haploid parental strains D7 and D7B, was employed to simultaneously determine cytotoxicity, genotoxicity (mitotic gene conversion and reverse mutation) and antimutagenesis of different substances [40].

Toxicity of dimethyl sulfoxide (DMSO) and *C. maritimum* was preliminarily tested. No significant cytotoxic or mutagenic effects were detected at concentrations ranging from 0.03 to 0.12 mg/mL compared to the control (CTR) both in incubation and in growth experiments. The treatment with hydrogen peroxide (H_2_O_2_) and with manadione (ME) caused a dose-dependent survival decrease from 4 mM (50%) to 40 mM for H_2_O_2_ and from 0.15 mM (50%) to 0.4 mM for ME (data not shown).

**Figure 5:**
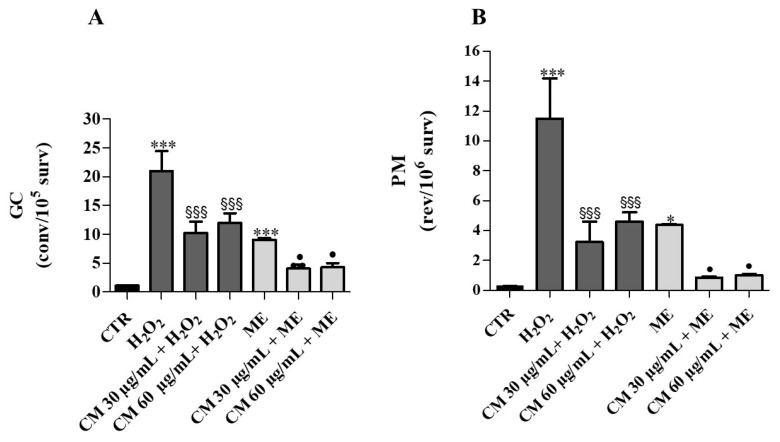
Incubation assay: Effects of *C. maritimum* L. extract (CM) (30 and 60 μg/mL) on *Saccharomyces cerevisiae* yeast exposed to an oxidative insult by H_2_0_2_ (4 mM) and ME (0.15 mM). (**A**), GC frequency expressed as convertants/10^5^ survivors; (**B**), PM frequency expressed as revertants/10^6^ survivors. Values are expressed as the mean ± SD of three independent experiments and analyzed by one-way ANOVA with Tukey multiple comparison test. Significant difference from CTR: * *p* ≤ 0.05; *** *p* ≤ 0.001. Significant difference from H_2_O_2_: **^§§§^** *p* ≤ 0.001. Significant difference from ME: **^•^** *p* ≤ 0.05.

Mutagenicity study during incubation assay showed that H_2_O_2_ (4 mM) and ME (0.15 mM) caused a 20-fold and 9-fold increase of mitotic gene conversion (GC), respectively, with respect to control (Figure 5A). After treating cells with *C. maritimum* and H_2_O_2_, a significant reduction (50%) in GC was evidenced, indicating a protective action of sea fennel extract against oxidative damage caused by H_2_O_2_. Similar observations could be made upon ME and sea fennel co-treatment. *C. maritimum* extract also mitigated GC upon H_2_O_2_ + ME treatment. Similarly, the treatment with H_2_O_2_ or ME significantly increased the point reverse mutation (PM) values (by 38-fold and 15-fold, respectively) compared to control, and *C. maritimum* was able to significantly reduce this mutagenic effect (Figure 5B).

**Figure 6:**
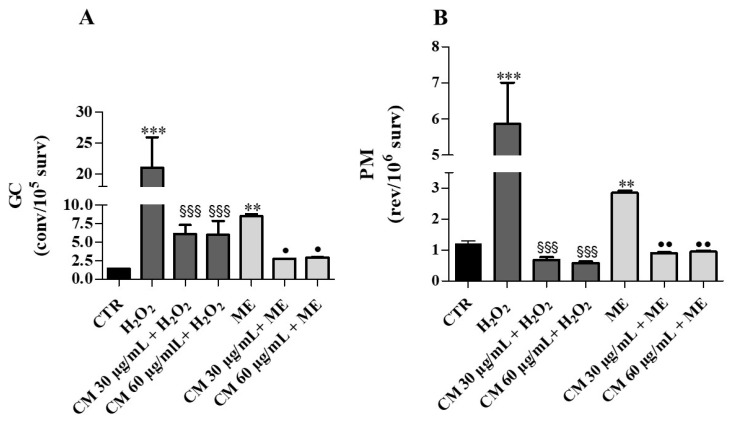
Growth assay: Effects of *C. maritimum* L. extract (CM) (30 and 60 μg/mL) on *Saccharomyces cerevisiae* yeast exposed to an oxidative insult by H_2_0_2_ (4 mM) and ME (0.15 mM). (**A**), GC frequency expressed as convertants/10^5^ survivors; (**B**), PM frequency expressed as revertants/10^6^ survivors. Values are expressed as the mean ± SD of three independent experiments, and are analyzed by one–way ANOVA with Tukey multiple comparison test. Significant difference from CTR: ** *p* ≤ 0.01, *** *p* ≤ 0.001. Significant difference from H_2_O_2_: **^§§§^** *p* ≤ 0.001. Significant difference from ME: **^•^** *p* ≤ 0.05, **^••^** *p* ≤ 0.01.

Mutagenicity study during growth assay showed that treatments with H_2_O_2_ and ME caused 15-fold and 6-fold increases of GC, respectively (Figure 6A). In the cells grown in the presence of *C. maritimum* extract, H_2_O_2_ or ME treatment induced about 3-fold less GC (compared to H_2_O_2_ or ME treatment alone). These results confirm the protective action of sea fennel extract against the genotoxicity induced by H_2_O_2_ and ME. Furthermore, H_2_O_2_ and ME caused significant increases of revertants (by about 5-fold and 2-fold, respectively) compared to control. *C. maritimum* was able to significantly reduce the PM frequency caused by H_2_O_2_ and ME (Figure 6B).

Regarding the two different experimental procedures, we can assert that the hydro-ethanolic *C. maritimum* extract has demonstrated strong antimutagen effects. Moreover, it was more efficient in growth experiments, preventing mutation induced by the oxidative damage caused by H_2_O_2_. 

## 3. Materials and Methods

### 3.1. Plant Material

Leaves of *Crithmum maritimum* L. were identified and sampled on rocks along the shoreline at “Sainte Anne du Portzic” by Professor Christian Magné (Brittany, France). The leaves were cleaned with deionized water, rapidly soaked, stored at −20 ℃ and then freeze-dried. The dry material was ground to a fine powder and suitably stored until analysis.

### 3.2. Hydro-Ethanolic Extraction

One gram of sea fennel leaf powder was macerated in 10 mL of 80% ethanol overnight under stirring at room temperature. The mixture was centrifuged for 10 min at 3500× *g* at 4 ℃ (Jouan CR3i centrifuge, Newport Pagnell, UK) and supernatant was collected, filtered (0.2 mm VWR International PBI, Milan, Italy) and kept at 4 °C in the dark until use. The extraction was repeated twice on the pellet and the three filtered supernatants were gathered. This procedure resulted in a *C. maritimum* extract with a yield of 25%.

### 3.3. Phytochemical Characterization and Phenolic Compounds Profiling by LC-ESI-MS Analysis

#### 3.3.1. Phenolic Contents of *C. maritimum* L. Extract 

The total phenolic content was determined by the Folin Ciocalteu colorimetric method [41] and expressed as mg of gallic acid equivalents/g dry weight (mg GAE/g DW). The total flavonoid contents were quantified using the aluminium chloride colorimetric method [42] and expressed as mg catechin equivalent (CE)/g DW. The total flavonols were measured according to the method described by Romani et al. [43] and expressed as mg quercetin equivalent (QE)/g DW. The total condensed tannins were measured using the modified vanillin assay described by Sun et al. [44] and expressed as mg catechin equivalent (CE)/g DW.

#### 3.3.2. Phytochemical Profile of *C. maritimum* L. Extract

The phenolic profile of *C. maritimum* extract was characterized by a Shimadzu UFLC XR system (Shimadzu, Kyoto, Japan), equipped with a SIL-20AXR auto-sampler, a system controller SCL-10A, a CTO-20 AC column oven, an LC-20ADXR binary pump, and a quadripole 2020 detector system. This instrument was equipped with a Discovery BIO Wide Pore C18 column (S250 × 4.0 mm id; 5 µm). The column temperature was set at 40 °C and the injection volume was 5 µL with a flow rate of 0.5 mL/min. Water with 0.1% formic acid and methanol with 0.1% formic acid were used as mobile phases A and B, respectively. The analysis was carried out using a linear gradient programmed as follows: 0–14 min, from 10% to 20% B; 14–27 min, from 20% to 55% B; 27–37 min, from 55% to 100% B; 37–45 min, 100% B; and 45–50 min 10% B. The conditions used for MS with the electrospray ionization (ESI) source were: dissolving line temperature was 280 °C, nebulizing gas flow 1.50 L/min and the drying gas, nitrogen, was set at 15.0 L/min. LC-ESI (-) MS mass spectra [M-H]- were acquired using LabSolutions software (Shimadzu). The authentic compounds were used as internal standards to identify and quantify the individual phenolic acids and flavonoids and also by comparision with retention time and mass spectra of authentic standards run using the same conditions (quinic acid, gallic acid, protocatechuic acid, chlorogenic acid, caffeic acid, syringic acid, *ρ*-coumaric acid, trans-ferulic acid, *O*-coumaric acid, *trans*-cinnamic acid, 4-*O*-caffeoylquinic acid, 1,3-di-*O*-caffeoyl-quinic acid, 3,4-di-*O*-caffeoylquinic acid, 4,5-di-*O*-caffeoylquinic acid, rosmarinic acid, salvianolic acid, (+)-catechin, epicatechin, acacetin, apigenin-7-*O*-glucoside, apigenin, quercitrin, kaempferol, cirsilineol, cirsiliol, quercetin-3-*O*-galactoside, luteolin-7-*O*-glucoside, luteolin, naringenin, naringin, quercetin-3-*O*-rhamnoside, rutin, and silymarin).

### 3.4. Antioxidant Activities (DPPH, ORAC and FRAP)

The scavenging activity on DPPH radicals of ethanolic extracts of *C. maritimum* leaves was determined following the method reported by Sokmen et al. [45]. The oxygen radical absorbance capacity (ORAC) of *C. maritimum* leaf extract was evaluated according to the method reported by Bacchiocca et al. [46] with some modifications as described by Gabriele et al. [47]. The ferric reducing antioxidant power (FRAP) was measured according to the method reported by Rodrigues et al. [48].

### 3.5. Preparation of Erythrocytes

Human blood samples from healthy volunteers were obtained upon informed consent for the use of residual blood for research purposes according to the Italian regulations and, in particular, the regulations of “Fondazione G. Monasterio CNR-Regione Toscana”. Blood samples were collected in EDTA-treated tubes and centrifuged 2300× *g* for 10 min at 4 °C. Plasma and buffy coat were discarded.

#### 3.5.1. Cellular Antioxidant Activity (CAA) in Human Erythrocytes

The antioxidant activity of increasing concentrations of *C. maritimum* extract (0.05, 0.1 and 0.2 mg/mL) was evaluated in an ex vivo erythrocytes system under oxidative condition according to the method described by Frassinetti et al. [49]. The fluorescence generated upon oxidation of DCFH-DA to highly fluorescent DCF by AAPH radicals was read at 485 nm excitation and 535 nm emission by using a VictorTM X3 Multilabel Plate Reader (Perkin Elmer, Waltham, MA, USA). Each value was expressed as follows: CAA unit = 100 − (∫SA/∫CA) × 100, where ∫SA is the integrated area under the sample curve, and ∫CA is the integrated area under the control curve (AAPH only-treated cells).

#### 3.5.2. Oxidative Erythrocytes Hemolysis

The anti-hemolytic effect of increasing concentrations of *C. maritimum* extract (0.05, 0.1 and 0.2 mg/mL) was determined according to Frassinetti et al. [50]. Oxidative erythrocytes hemolysis was generated by thermal decomposition of AAPH in peroxyl radicals. A control and blank sample were used and refer to erythrocytes exposed to AAPH or PBS, respectively. Quercetin was used as an antioxidant standard. The erythrocytes hemolysis was spectrophotometrically evaluated at 540 nm as hemoglobin released in supernatant. Each value was expressed as a percentage of hemolysis with respect to the control (AAPH only-treated erythrocytes).

### 3.6. Antimicrobial Activity

#### 3.6.1. Bacterial Media

Mueller Hinton Broth (MHB), Mueller Hinton Agar (MHA), Mc Farland standard 0.5, Tripticase Soy Broth (TSB), were purchased from Oxoid (Basingstone, UK).

#### 3.6.2. Growth Conditions of Pathogenic Bacteria

The pathogenic bacterial strains were supplied by the American Type Culture Collection (ATCC, Manassas, USA, Virginia). The antimicrobial activity of hydro-ethanolic *C. maritimum* extract was studied on three Gram negative bacteria, specifically *Escherichia coli* (ATCC 25922), *Salmonella enterica* ser. *typhimurium* (ATCC 14028), and *Enterobacter aerogenes* (ATCC 13048), and two Gram positive bacteria, *Enterococcus faecalis* (ATCC 29212) and *Staphylococcus aureus* (ATCC 25923). All bacteria strains were grown on MHB and incubated overnight at 37 °C under aerobic conditions.

#### 3.6.3. Antimicrobial Activity

The final O.D. of selected bacteria incubated with hydro-ethanolic *C. maritimum* extract was determined according to Delgado et al. [14], with some modifications. *C. maritimum* leaves extracts were diluted in sterile water to the concentration of 1 mg/mL, then further dilutions were made up to the concentration of 0.05 mg/mL.

The tested bacteria were cultured in Mueller Hinton Broth (MHB) at 37 °C for 16 h and diluted to match the turbidity of 0.5 McFarland unit. An aliquot of 50 µL of bacterial suspensions (about 1–5 × 10^5^ CFU/mL) was added to 100 µL of MHB and to 100 µL of hydro-ethanolic *C. maritimum* extract (0.25, 0.50 and 1 mg/mL) in a 96-well plate. A negative control of 100 µL of water was included on each microplate. The plates were incubated at 37 °C for 24 h in aerobic conditions. Afterwards, the final optical density (O.D.) at 600 nm was determined by a microplate reader (Eti-System Fast Reader Sorin Biomedica, Modena, Italy).

#### 3.6.4. Biofilm Production and Inhibition (Crystal Violet Assay)

The biofilm production was determined using the method described by Di Ciccio et al. [49] with some modifications as described by Blando et al. [51]. The assay was performed using two *Staphylococcus* strains: the biofilm producer *Staphylococcus aureus* (ATCC 35556) and *Staphylococcus epidermidis* (ATCC 12228), used as a negative control, since it does not produce biofilm. The strains were activated by culturing in 5 mL of TSB at 37 °C for 24 h. After 24 h, the absorbance was measured at 600 nm and appropriate dilutions were made in TSB + 1% sucrose, to obtain an optical density of 0.1 corresponding to about 10^6^ cells/mL. The assay was performed in sterile 96-well polystyrene plate (Greiner Bio-One GmbH, Kremsmünster, Austria). Briefly, 100 µL of *Staphylococcus aureus* suspension were inoculated and cultured with or without 100 µL of *C. maritimum* leaves extract (at concentrations ranging from 0.1 to 1 mg/mL), without shaking at 37 °C. After 24 h incubation, non-adherent cells were removed by dipping each sample three times in sterile PBS. Samples were fixed at 60 °C for 1 h and the biofilms were stained with 0.1% solution of crystal violet in water, according to O’Toole [52]. After staining, samples were washed thrice with distilled water. The quantitative analysis of biofilm production was performed by adding 125 µL of 30% acetic acid to de-stain the samples. Afterwards, the absorbance was measured at 492 nm using the microplate reader. The percentage of biofilm inhibition was determined by the formula:

$$\mathrm{Biofilm}\;\mathrm{reduction}\;\left(\%\right)=\frac{\mathrm{Acontrol}-\mathrm{Asample}}{\mathrm{Acontrol}}\times 100$$

### 3.7. Antimutagenesis Assay in Yeast Cells

The hydro-ethanolic *C. maritimum* extract was evaporated under *vacuum* (yield 22%) and the pellet was dissolved in DMSO (55 mg/mL). Yeast cells (D7 strain) from a standard culture were incubated in liquid medium containing 2% glucose under shaking (30 °C). There are several experimental methodologies for carrying out genotoxicity tests in yeast *Saccharomyces cerevisiae* to evaluate the potential effect of a compound and in this work we decided to employ cells in logarithmic phase because they are metabolically active [53]. About 15 × 10^5^ cells/100 mL of liquid medium were incubated *over-night* under shaking (30 °C) until logarithmic phase was reached (70–90 × 10^6^ cells/mL), with or without *C. maritimum* extract.

Two different experimental procedures were followed:

(1) Incubation assay: the *C. maritimum* extract (30 and 60 µg/mL) and oxidizing agents were incubated for 90 min, under shaking (30 °C) together with cells in logarithmic phase.
(2) Growth assay: the oxidizing substances were added in the flasks where yeast cells were grown together with *C. maritimum* and incubated for 90 min under shaking (30 °C).

H_2_O_2_ and ME were used as oxidizing agents because, as reported in the literature, both of them cause oxidative stress in cells with two different mechanisms of action. H_2_O_2_ is reduced by ions metals through the Fenton reaction with the consequent production of hydroxyl radicals, whereas ME forms H_2_O_2_ and superoxide radicals along with semiquinones [54]. Before setting up antimutagenesis tests to evaluate the mutagenic and/or potential protective effect of *C. maritimum*, toxicity tests were performed.

### 3.8. Statistical Analysis

Assays were carried out in triplicate and results were expressed as mean values ± standard deviation (SD). Data were analyzed by one-way analysis of variance (ANOVA) with Dunnett’s and Tukey multiple comparison test (GraphPad Prism software, version 6.00 for Windows, GraphPad Software Inc., San Diego, CA, USA). A *p* < 0.05 was considered statistically significant.

## 4. Conclusions

Our study confirmed that the hydro-ethanolic extract of *C. maritimum* leaves contains plentiful soluble polyphenols with high quantitative and qualitative variability. These bioactive components exhibited a strong antioxidant property as demonstrated by their high DPPH, ORAC and FRAP-scavenging activities. The phytochemical profile using LC-ESI-MS allowed us to identify eighteen compounds, mainly chlorogenic acids and flavonoids, among which cirsiliol (a flavonol) was identified for the first time in *C. maritimum*. As a consequence of its richness in phenolic compounds, sea fennel extract exhibited strong antioxidant activities, and a dose-dependent protection from oxidative hemolysis. In addition, *C. maritimum* extract showed a strong antibacterial activity and inhibited biofilm formation. Although interesting results were obtained for both mutagens, *C. maritimum* extract has demonstrated greater efficacy against the oxidative damage produced by H_2_O_2_ for PM induction in growth experiments. Results demonstrated that the presence of bioactive compounds in the extract are more effective at the beginning of growth, preparing the cells for the insult of the oxidant. This work confirms literature data, suggesting that protection is due to the presence of antioxidants, especially phenolic acids. The results obtained in this study indicate that *C. maritimum* hydro-ethanolic leaves extract are rich in beneficial bioactive compounds, possess in vitro and ex vivo antioxidant activity, and also antimutagenic activity on yeast *S. cerevisiae* and they can be used for food purposes. 

## Figures and Tables

**Table 1:** Content of phenolic classes and in vitro antioxidant activities of hydro-ethanolic extract of *C. maritimum* leaves.

*C. maritimum* Leaves Extract	
Total polyphenols (mg GAE g^−1^ DW)	31.70 ± 0.09
Total flavonoids (mg CE g^−1^ DW)	25.61 ± 0.04
Flavonols (mg QE g^−1^ DW)	17.30 ± 0.06
CTC (mg CE g^−1^ DW)	0.97 ± 0.09
DPPH (IC_50_, mg mL^−1^)	0.22 ± 0.04
ORAC (µmol TE g^−1^ DW)	15,835 ± 71
FRAP (EC_50_, mg mL^−1^)	1.82 ± 0.02

CTC, Condensed tannin contents; ORAC, Oxygen radical absorbance capacity; FRAP, Ferric reducing antioxidant power.

**Table 2:** Content of phenolic acids, flavonoids and flavonols (mg g^−1^ DW) identified in the hydro-ethanolic extract of *C. maritimum* leaves by LC-ESI-MS.

**N°**	**Metabolic Class Subclass**	**RT (min)**	**Compound Identified**	Content (mg g^−1^ DW)
5	Organic acid Phenolic acids Hydroxycinnamic acids	8.2	Quinic acid	3.78
1		5.0	Gallic acid	-
2		11.1	Chlorogenic acid	7.25
3		25.8	Neochlorogenic acid	2.03
4		23.9	Cryptochlorogenic acid	1.17
6		23.0	Caffeic acid	0.67
7		20.3	p-Coumaric acid	1.02
8		27.7	Trans ferulic acid	1.41
9		24.9	4,5-di-*O*-caffeoylquinic acid	0.72
	Flavonoids			
10		24.4	Naringin	0.14
11		40.5	Naringenin	0.09
12		42.8	Luteolin-7-*O*-glucoside	0.35
13		18.2	Rutin	1.75
14		35.5	Cirsiliol	1.31
	Flavonols			
15		25.4	Quercetin	0.09
16		23.7	Kaempferol	0.72
17		35.0	Hyperoside (quercetin-3-*O*-galactoside)	1.12
18		19.8	Quercetrin (quercetin-3-*O*-rhamnoside)	1.41
	Total			25.03

## Data Availability

The data presented in this study are available on request from the corresponding author.

## Sample Availability

Samples of the compounds are not available from the authors.
